# Patients with Orthostatic Intolerance: Relationship to Autonomic Function Tests results and Reproducibility of Symptoms on Tilt

**DOI:** 10.1038/s41598-017-05668-4

**Published:** 2017-07-18

**Authors:** Hyung Lee, Phillip A. Low, Hyun Ah Kim

**Affiliations:** 1 Department of Neurology, Keimyung University School of Medicine, Daegu, South Korea; 2 Brain Research Institute, Keimyung University School of Medicine, Daegu, South Korea; 3 Department of Neurology, Mayo Clinic, Rochester, Minnesota USA

## Abstract

This study was designed to investigate the frequency and pattern of orthostatic symptoms during head-up tilt (HUT) in patients with orthostatic intolerance during daily life, and to identify the relationship between the orthostatic symptoms during HUT and autonomic parameters. We prospectively collected autonomic data from 464 patients with orthostatic symptoms. Adrenergic and cardiovagal function tests including HUT were performed. Based on HUT results, we divided patients into orthostatic hypotension (OH), postural tachycardia syndrome (POTS), or normal HUT groups. We also investigated orthostatic symptoms during HUT. Only 25% of the patients reported orthostatic symptoms during HUT and 75% were asymptomatic. Typical orthostatic symptoms such as orthostatic dizziness and blurred vision, and atypical symptoms like chest tightness and headache occurred in 86% and 66%, respectively. Patients with POTS had symptoms more frequently than patients with OH during HUT. There were no differences in degrees of BP or HR changes between symptomatic and asymptomatic groups within the OH and POTS groups. HUT fails to reproduce symptoms of orthostatic intolerance in the majority of patients. Clinicians need to be aware that most patients with OH are asymptomatic during HUT and patients with POTS are more likely to be symptomatic than patients with OH.

## Introduction

Orthostatic symptoms are defined as symptoms that occur when upright and are relieved by recumbence. Common orthostatic symptoms are dizziness, lightheadedness, weakness or tiredness, cognitive difficulties, blurred vision, anxiety, tremulousness, palpitations, nausea, clamminess, or sweating^1, 2^. Two orthostatic intolerance syndromes, orthostatic hypotension (OH) and the postural tachycardia syndrome (POTS), are thought to be responsible for these symptoms. Head-up tilt (HUT) is a standard test to evaluate orthostatic intolerance. However, even in a well-defined group of patients with orthostatic symptoms and documented OH, reproducibility of OH with HUT is relatively low^3^. Furthermore, patients with prominent OH during HUT can have no symptom during daily life (i.e., asymptomatic OH)^1^ or during HUT (i.e., hypotension unawareness)^4^. This discordance may relate to multiple mechanisms causing OH and the origin of orthostatic intolerance. Hence, we undertook a study with the following goals. First, we undertook a battery of autonomic function tests to separate these patients into categories of OH, POTS, and normal autonomic function. Second, we evaluated the ability of HUT to reproduce symptoms of orthostatic intolerance. Third, we evaluated if symptoms related to severity of autonomic failure.

## Methods

We prospectively collected data from a series of consecutive patients with orthostatic symptoms who were seen in the Dongsan Medical Center Autonomic Reflex Laboratory from March 2014 to June 2015. We included all patients who experienced orthostatic symptoms while they were sitting or standing within the previous 6 months. We excluded patients under 10 years old. A standardized battery of adrenergic autonomic tests, including HUT, Valsalva maneuver (VM) and heart rate (HR) response to deep breathing (HRDB) test using Finometer devices (FMS, Amsterdam, The Netherlands) for recording the beat-to-beat BP and HR response, was performed in all patients according to a previously validated method for the diagnosis of autonomic dysfunction^1^. In addition to BP monitoring using Finometer, BP was obtained at baseline and every minute during the tilt using a manual sphygmomanometer (Tycos, Skaneateles Falls, NY, USA). Before autonomic function tests, all patients were asked to complete the Korean version of the orthostatic grading scale (KOGS), which is a validated tool for screening patients with orthostatic dizziness during daily life in Korea^5^.

Based on HUT, we divided patients into orthostatic hypotension (OH), POTS, or normal HUT groups. The tilt protocol included at least 10 min in the supine position and 20 min of a tilt at 70 degrees. OH was defined by a decrease in systolic BP (SBP) of at least 20 mmHg or a decrease in diastolic DBP (DBP) of at least 10 mmHg between supine rest for 10 min and an upright posture for 20 min^6^. OT was defined as a sustained HR increment of 30 beats per minute within 10 min of HUT in the absence of OH. For individuals aged under 19 years, the required increment is at least 40 beats/minute^6^. The reflex syncope was not included in our study. “Normal HUT group” was defined as a group of patients with previous history of orthostatic symptoms, but failed to develop orthostatic symptoms on HUT. We also investigated the orthostatic symptoms during HUT and divided the patients into 2 groups: symptomatic group (i.e., orthostatic symptom during HUT) and asymptomatic group (i.e., no symptoms during HUT). We made a list of orthostatic symptoms including dizziness or lightheadedness, blurred vision, chest tightness, shortness of breath, pain in the legs or back, nausea, cold sweating, tingling sensation on legs, headache, palpitation, tremor, tiredness, and cognitive problems. We asked to patients after HUT if the patient experienced symptoms on the list and allowed them to report other symptoms as well.

For VM, patients were instructed to take a deep breath and blow into a syringe through a mouthpiece attached to a manometer for 15 s until expiratory pressure was reached to 40 mmHg. BP magnitude was determined for four phases, i.e., phase I, early and late phase II, phase III, and phase IV as previously described^1, 7^. A maximal drop in the mean BP (MBP) of more than 20 mmHg during early phase II was abnormal^8^. Late phase II was considered abnormal if the magnitude of the end of phase II did not exceed the baseline (i.e., a negative value) except men beyond the age of 60 years^8, 9^. Phase IV was considered abnormal if the magnitude of the phase IV response failed to reach the baseline (i.e., a negative value)^8, 10^. The difference in BP between the baseline and the end of phase II, and pressure recovery time (PRT) were calculated and determined as abnormalities from VM results^10^. According to the PRT results, we defined neurogenic OH if patients fulfilling OH who also had an abnormally prolonged PRT (≥5 seconds)^11^. A “flat-top” response, which indicated that the mean BP did not fall below baseline levels during phase II, was regarded as an incomplete study. The cardiovagal function was evaluated by the expiration (E): inspiration (I) ratio, the HR response to deep breathing (HR_DB_), and Valsalva ratio (VR) during VM.

We excluded patients who were receiving medications with potential effects on autonomic function, such as beta-blockers, anticholinergic agents, and antihypertensive drugs. All of the experiments complied with the tenets of the Declaration of Helsinki, and the study protocol was also reviewed and approved by Keimyung University Institutional Review Board. Informed consent was obtained from all participants.

We compared the clinical characteristics and autonomic test results between symptomatic and asymptomatic group with the independent-samples t-test for continuous variables and chi-squared test for categorical variables. We used Levene’s test for analysis of homoscedasticity for continuous variables. We calculated total statistical power and effect size and denied the statistical significance of the comparison if the power was not enough. One-way ANOVA and Tukey test for post hoc analysis were used to compare continuous variables between the OH, POTS and normal HUT groups. The mean value ± standard deviation (SD) is displayed. A probability of p < 0.05 was considered to be statistically significant.

## Results

We identified 464 patients (237 men and 227 women) with autonomic symptoms suggesting orthostatic intolerance who performed autonomic function tests including HUT. The average age of the patients was 49.8 ± 22.27 years with a range from 11 to 92 years. We analyzed data from HUT result in all 464 patients and VM test result in 395 patients. One hundred three patients had hypertension, 75 diabetes, 52 stroke, 30 degenerative diseases such as Parkinson’s disease, multiple system atrophy or Alzheimer’s dementia, 25 polyneuropathy, 34 heart disease, 19 epilepsy, 12 thyroid disease, 8 cancer, 2 chronic renal failure, 3 fibromyalgia, and 1 spinal cord injury. Fifty-six percent of the patients (260/464) had no previous medical illness. Mean total KOGS score during an ordinary life was 5.6 ± 2.6 (range 1~15).

Only twenty-five percent of the patients (116/464) were symptomatic during HUT; the others (75%) denied any symptoms during HUT. The mean onset time of orthostatic symptom was 5.0 ± 4.8 minutes after commencement of HUT and the mean duration of orthostatic symptoms was 10.7 ± 8.0 minutes. Approximately 30% (31/116) of the symptomatic group failed to continue HUT because of intolerable symptoms or presyncope. Typical orthostatic symptoms, such as orthostatic dizziness (i.e., lightheadedness) (72, 62%), blurred vision (26, 22%), nausea (26, 22%), cognitive problem (22, 19%), palpitation (25, 22%), cold sweating (15, 13%), and tiredness (14, 12%), were occurred in 86% (100/116) of symptomatic patients during HUT. Atypical orthostatic symptoms, such as chest tightness (52, 43%), headache (14, 12%), leg pain (13, 11%), difficulty of breathing (14, 12%), tingling sensation on leg (8, 7%), and back pain (6, 5%) were reported in 66% (76/116) of symptomatic patients. Thirty-five percent of patients (40/116) reported only typical symptoms, 14% (16/116) had only atypical symptoms, and the other 52% of patients had both.

**Figure 1:**
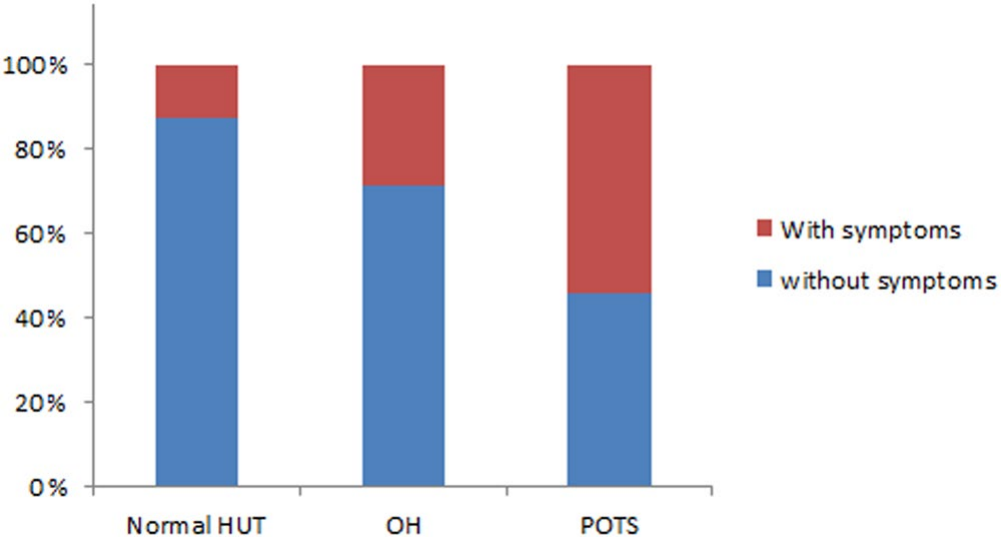
A proportion of patients with and without symptoms during the head-up tilt test in patients with orthostatic hypotension, orthostatic tachycardia and normal results in head-up tilt tests. HUT = head-up tilt test; OH = orthostatic hypotension; POTS = postural tachycardia syndrome.

**Figure 2:**
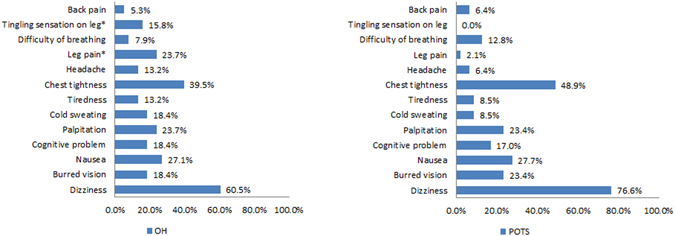
The frequency of orthostatic symptoms in patients with orthostatic hypotension (OH), postural tachycardia syndrome (POTS) in the head-up tilt tests.

Forty seven percent of the patients (219/464) showed changes in BP or HR during HUT that fulfilled criteria for OH or POTS. Twenty eight percent (132/464) of patients showed OH and 19% (87/464) POTS, and the others had normal results on HUT. Among the OH group, 53% (60/113) of patients had neurogenic OH and the others had non-neurogenic OH. We excluded 19/132 patients with recordings that were technically not suitable for analysis, primarily “flat-top” responses. There were significant differences in age, hemodynamic and autonomic parameters in standardized autonomic function tests among the three groups (Table s1). The OH group had changes in both adrenergic and cardiovagal parameters during HUT, VM and HRDB. The POTS group showed higher E:I ratio, HR_DB_ and VR compared to the normal HUT group. Patients with POTS had orthostatic symptoms more frequently during HUT compared to patients with OH (47/87, 54.0% vs. 38/132, 28.8%, p < 0.001). There was no difference of frequency of symptoms between neurogenic (14/60, 23.3%) and non-neurogenic (18/53, 34%) OH group (p = 0.296). Thirteen percent (31/245) of the patients with normal HUT results had orthostatic symptoms during HUT (Fig. 1). Among orthostatic symptoms during HUT, patients with OH showed a higher frequency of leg pain (23.7% vs. 2.1%, p = 0.004), and tingling sensation (15.8% vs. 0%, p = 0.006) during HUT than in the patients with OH. There were no differences of frequency of symptoms due to cerebral hypoperfusion including dizziness and blurred vision, and cerebral activation such as palpitation, tremulousness, or nausea between the OH and POTS groups (Fig. 2).

Symptomatic group was younger than the asymptomatic group in total population groups (41.69 ± 22.62 vs. 52.44 ± 21.52, p < 0.001). There were significant differences in hemodynamic and autonomic parameters between symptomatic and asymptomatic groups. Baseline DBP, maximum decrease of SBP and increase of HR during HUT, increase of MBP in Phase IIL and difference in BP between the baseline and the end of phase II in VM were higher in symptomatic group. Symptomatic group also showed higher total KOGS scores than asymptomatic group (Table 1). However, inside the OH and POTS group, there were no significant differences in age, hemodynamic and autonomic parameters during HUT between symptomatic and asymptomatic groups. In the normal HUT group, symptomatic patients showed higher total KOGS scores than asymptomatic group (Table 1).

**Table 1:** Comparison of autonomic parameters in patients with and without orthostatic symptoms during head-up tilt test.

Tests	Parameter	Total				OH				POTS				Normal HUT results			
		Without symptoms (n = 348)		With symptoms (n = 116)		Without symptoms (n = 94)		With symptoms (n = 38)		Without symptoms (n = 40)		With symptoms (n = 47)		Without symptoms (n = 214)		With symptoms (n = 31)	
		Mean	SD	Mean	SD	Mean	SD	Mean	SD	Mean	SD	Mean	SD	Mean	SD	Mean	SD
HUT	Baseline SBP (mmHg)	129.1	23.0	133.1	21.9	130.6	23.6	140.4	25.5	122.9	13.9	128.2	14.5	129.6	23.9	131.7	24.5
	Baseline DBP (mmHg)	64.7*^b^	9.6	68.6*^b^	9.0	64.9	9.9	68.9	9.1	68.9	6.0	71.0	7.4	63.8	9.8	64.6	9.8
	Baseline HR (bpm)	65.0	11.6	67.6	12.2	65.9	11.9	67.0	10.7	68.1	10.3	69.1	11.7	64.1	11.6	66.0	14.6
	Decrease in maximum SBP (mmHg)	17.4*^a^	13.8	22.0*^a^	16.7	33.9	10.9	37.5	16.2	14.0	9.0	18.0	9.1	10.8	8.8	9.2	11.2
	Decrease in maximum DBP (mmHg)	6.0	7.1	7.3	10.5	13.6	7.1	15.1	11.2	1.2	6.0	4.7	8.7	3.5	4.2	1.7	5.3
	Increase in maximum HR (bpm)	17.3*^c^	10.6	26.1*^c^	14.0	14.2	7.6	17.0	10.7	39.5	7.1	39.4	8.5	14.5	6.7	16.9	5.9
VM	Fall of MBP in Phase IIE (mmHg)	11.6	8.0	11.2	7.5	14.0	9.6	14.7	8.7	10.7	8.8	9.9	5.6	10.7	6.9	11.5	8.4
	Increase of MBP in Phase IIL (mmHg)	12.6*^a^	12.5	15.9*^a^	12.4	5.1	12.2	8.7	12.5	19.7	11.6	20.1	9.1	14.6	11.2	18.2	13.2
	Difference in BP between the baseline and the end of phase II (mmHg)	0.8*^a^	14.5	4.7*^a^	13.6	8.9	16.8	4.0	15.5	8.0	9.7	10.2	8.1	3.8	11.9	6.6	12.9
	Increase of MBP in Phase IV (mmHg)	13.6	12.9	14.7	13.3	7.9	12.4	8.2	10.6	21.6	13.8	18.8	13.5	14.6	11.9	16.2	13.4
	PRT (second)	4.6	5.7	3.6	4.8	8.8	8.2	6.2	6.0	1.7	1.0	1.9	3.1	3.2	3.6	3.0	4.2
Total KOGS score		5.3*^b^	2.4	6.4*^b^	2.8	5.4	2.8	5.6	2.1	5.8	3.3	7.2	3.2	5.2*^b^	2.0	6.5*^b^	3.0

SD = standard deviation; HUT = head-up tilt test; VM = Valsalva maneuver; HR = heart rate; SBP = systolic blood pressure; DBP = diastolic blood pressure; MBP = mean blood pressure; PRT = pressure recovery time; Phase IIE = early phase II; Phase IIL = late phase II; KOGS = Korean version of the orthostatic grading scale; OH = orthostatic hypotension; POTS = postural orthostatic tachycardia syndrome. *Significant between symptomatic and asymptomatic group (p < 0.05, total power > 80%); independent t-test. ^a^Small effect size in Cohen’s standard. ^b^Moderate effect size in Cohen’s standard. ^c^Large effect size in Cohen’s standard.

We included patients with a BP increase during HUT in the normal HUT group rather classified them into a new group with orthostatic hypertension. There were 15 patients (15/464, 3.2%) with orthostatic BP increase over 20 mmHg during HUT and three of them (3/15, 20%) were symptomatic during tilting. After excluding patients with orthostatic hypertension from the normal HUT group, 12.2% (28/230) patients with normal HUT results had symptoms during HUT.

## Discussion

There were 2 main findings of our study. First, HUT failed to reproduce symptoms of orthostatic intolerance in the majority of patients. Second, POTS patients, without autonomic failure, were more likely to be symptomatic than OH patients, who usually have autonomic failure. Our study comprised a good mix of patients with orthostatic dizziness seen in practice. The clear message is that symptoms are a poor predictor of autonomic abnormalities. First of all, HUT reproduced symptom in only one-fourth of patients. Second, only one-half demonstrated abnormalities of BP or heart rate in autonomic function testing. This value of 25% reproduction of symptoms with HUT is lower than the previously reported frequency (67%) of orthostatic symptoms during the tilt^4^. The disparity likely is due to the earlier report selectively focusing on the patients with profound OH (i.e., a decrease of SBP more than 60 mmHg) during HUT^4^. Although both studies focused on patients presenting various symptoms of orthostatic intolerance during daily life, there was a significant difference in terms of the inclusion criteria of the study group. In other studies, only 18.7% of OH patients and half of POTS patients were symptomatic during HUT^12^. These studies support our findings that symptoms of orthostatic intolerance are reproduced in small number of patients in HUT. The clinical relevance of our study lies in the finding that even patients who complain orthostatic symptoms do not necessarily have abnormal results in HUT and there is a possibility of the presence of hypotension or tachycardia unawareness.

Patients with OH often do not have symptoms, even with a large fall in BP. This OH unawareness is due to expansion of autoregulated range. Normal subjects have a small range of BP change where cerebral blood flow does not change within the autoregulated range. In patients with chronic OH, a significant expansion of this range, down to low BP as low as a systolic BP of 70 mm Hg can occur^13^. Awareness of HR increase has not been systematically studied. POTS patients with a heart rate increase >30 bpm typically have symptoms ascribed to cerebral hypoperfusion (such as lightheadedness) and sympathetic activation (such as palpitations and tremulousness). However, some patients do not have symptoms. Furthermore, some patients have significant orthostatic intolerance and do not have tachycardia. Such variability has been ascribed to changes in neural circuitry^14^ and to psychological mechanisms^15^, often described as somatic hypervigilance^16^. Although the term “tachycardia unawareness” has not previously been described, we found that half of the patients with POTS were asymptomatic during HUT. In previous studies, young normal controls showed excessive tachycardia over 30 bpm during tilt, but did not develop orthostatic symptoms^17, 18^. In follow up study, POTS patients showed improved orthostatic symptoms with still excessive tachycardia during the tilt in follow-up period^18^. Therefore, asymptomatic patient with excessive tachycardia during the tilt is not surprising and actually the cyclical nature of the symptoms is one of the features of POTS^16^. We used modified criteria for younger patients with POTS, but even so, tachycardia unawareness can still occur. The mechanism of tachycardia unawareness is not clear. In the present study, considering that all patients with tachycardia unawareness were symptomatic during daily life, it is possible that they may experience fluctuating symptoms because of changing conditions such as menstruation, mental stress, weight, or fluid changes even they have constant orthostatic tachycardia^16^. In addition to OH or POTS, we assured the presence of orthostatic hypertension during HUT in patients with orthostatic symptoms. We have previously reported that orthostatic intolerance due to orthostatic hypertension has been underestimated^19^. The prevalence of orthostatic hypertension in the present study was 3.2% and only 20% of them were symptomatic. Orthostatic hypertension also contributes to the gap between orthostatic symptoms and abnormalities of HR or HR by HUT and VM.

Similar to a previous study^4^, we found that patients commonly reported atypical orthostatic symptoms such as chest tightness as well as typical orthostatic symptoms such as postural dizziness. Our study confirmed the previous notion^4^ that atypical symptoms are almost as common as typical symptoms such as postural dizziness during HUT in patients with orthostatic intolerance during daily life. Thus, clinicians should consider the possibility of autonomic cause if symptoms occur during HUT, even in the absence of typical symptoms. Among atypical symptoms, leg pain and tingling sensation were more frequent in OH group compared to POTS group. There has been no report about the mechanism of leg pain and tingling sensation in patients with OH but this might be due to blood pooling by gravity in condition with vasoconstriction failure.

We found that changes in BP and HR during HUT were significantly more pronounced in the symptomatic group than in the asymptomatic group. The occurrence of orthostatic symptoms during the upright posture might closely be associated with the amount of changes in hemodynamic parameters such as BP and HR from the upright posture in all population groups. Indeed, OH and POTS are known to be a representative orthostatic intolerance syndrome that causes orthostatic symptoms such as dizziness. It has been well known that POTS and OH patients display autonomic impairment. OH patients have both vagal baroreflex failure as well as sympathetic adrenergic failure^20^. Patients with POTS usually present attenuated vagal baroreflex while sympathetic control is increased^21^. Our results showed definite baroreflex and adrenergic dysfunction of OH group compared to the normal HUT group, but cardiovagal parameters during HRDB and VM were higher than the normal HUT group in POTS group.

Within the OH and POTS groups, BP or HR changes during HUT were not significantly different between symptomatic and asymptomatic groups. In other word, we could not assume the severity of orthostatic symptoms during HUT as indicative of the degree of orthostatic fall in BP or HR rise during HUT. Moreover, the frequency of orthostatic symptoms during HUT between patients with neurogenic and non-neurogenic OH is not significantly different.

There has been debate around the optimal duration of tilt of HUT. Tilt duration is a more important variable than tilt angle and the duration of the tilt depends on the cause of orthostatic intolerance^22^. The duration of tilt is generally accepted that 5 minutes for orthostatic hypotension, 10 minutes for POTS and a longer duration for neurally mediated syncope^22^. To detect initial OH, we need to measure BP immediately after onset of tilt^23^. Delayed OH optimally requires 30 minutes in older individuals and 40 minutes required for younger individuals to detect^24^. For detecting late POTS, which occurred after 10 minutes of tilt, we need 45 minutes HUT^25^. According to the present study, we cannot predict the cause of orthostatic intolerance with symptoms. Therefore, clinicians need to carefully measure immediate BP and HR and to decide the duration of tilt according to BP and HR changes during HUT in addition to the suspected cause of orthostatic intolerance.

Our study had several limitations. One limitation is our criterion for OH. For tilt studies, requirement for a fall in SBP of >30 mm Hg might be more appropriate^26, 27^. By using the lower value we are including patients with mild autonomic failure. Second, we did not attempt to compare autonomic data from patients with orthostatic symptoms during daily life with that from the control group without orthostatic symptoms. Thus, the relevance of a single abnormal test result should be made with caution. Further studies, including a large number of normal controls, are needed to assess the clinical implication of mild degrees of autonomic dysfunction in each test. Third, we did not attempt to compare autonomic data from patients with typical symptoms with that of atypical symptomatic groups. Further studies are needed to assess the difference in autonomic parameters between patients with typical symptoms and patients with atypical symptoms. Finally, because we classified patients, according to the patterns of orthostatic symptoms, irrespective of their underlying conditions, the possible patterns of orthostatic symptoms according to underlying causes remained to be elucidated.

## Electronic supplementary material

Table S1
