# Supplementary material for: Patients with Orthostatic Intolerance: Relationship to Autonomic Function Tests results and Reproducibility of Symptoms on Tilt
Source: Sci Rep. 2017 Jul 18;7:5706. doi: 10.1038/s41598-017-05668-4 (PMC5515942; doi:10.1038/s41598-017-05668-4)
Supplement: Supplementary file 1 — Table S1 [file 41598_2017_5668_MOESM1_ESM.doc]

**Patients with Orthostatic Intolerance**

**: Relationship to Autonomic Function Tests results and Reproducibility of Symptoms on Tilt**

Hyung Lee MD^1,2^, Phillip A. Low MD^3^, Hyun Ah Kim MD^1,2^

Department of Neurology,^1^ Brain Research Institute^2^

Keimyung University School of Medicine, Daegu, South Korea

Department of Neurology, Mayo Clinic, Rochester, Minnesota, USA^3^

Supplementary Table S1. Comparison of autonomic function test results in patients with OH, POTS, and normal head-up tilt test results.

|  |  | OH (n=132) | | POTS (n=87) | | normal HUT (n=245) | |
| --- | --- | --- | --- | --- | --- | --- | --- |
|  |  | Mean | SD | Mean | SD | Mean | SD |
|  | Age^abc^ | 65.1 | 11.8 | 21.0 | 9.2 | 51.7 | 20.2 |
| HUT test | Baseline SBP (mmHg)^ab^ | 133.4 | 24.5 | 125.8 | 14.4 | 129.9 | 23.9 |
|  | Baseline DBP (mmHg)^ab, bc^ | 66.1 | 9.8 | 70.0 | 6.8 | 63.9 | 9.8 |
|  | Baseline HR (bpm)^ab, bc^ | 66.2 | 11.5 | 68.6 | 11.1 | 64.3 | 12.0 |
|  | Decrease in maximum SBP (mmHg)^abc^ | 34.9 | 12.7 | 16.1 | 9.2 | 10.6 | 9.2 |
|  | Decrease in maximum DBP (mmHg)^ab, ac^ | 14.0 | 8.5 | 3.1 | 7.8 | 3.3 | 4.4 |
|  | Increase in maximum HR (bpm)^ab, bc^ | 15.0 | 8.7 | 39.5 | 7.8 | 14.8 | 6.6 |
| Valsalva test | Fall of MBP in Phase IIE (mmHg)^ab, ac^ | 13.6 | 9.3 | 10.2 | 7.2 | 10.8 | 7.0 |
|  | Increase of MBP in Phase IIL (mmHg)^abc^ | 6.1 | 12.3 | 19.9 | 10.2 | 15.0 | 11.5 |
|  | Difference in BP between the baseline and the end of phase 2 (mmHg)^abc^ | 7.5 | 16.6 | 9.2 | 8.9 | 4.2 | 12.0 |
|  | Increase of MBP in Phase IV^abc^ | 8.0 | 11.9 | 20.1 | 13.7 | 14.8 | 12.1 |
|  | PRT (second)^ab, ac^ | 8.1 | 7.7 | 1.8 | 2.4 | 3.2 | 3.6 |
|  | Valsalva ratio ^abc^ | 1.4 | 0.3 | 2.0 | 0.4 | 1.6 | 0.3 |
| Heart rate deep breathing test | Heart rate response ^abc^ | 7.5 | 5.5 | 20.1 | 6.8 | 12.1 | 7.7 |
|  | E:I ratio ^abc^ | 1.1 | 0.1 | 1.3 | 0.1 | 1.2 | 0.1 |
| Total KOGS score^ab, bc^ | | 5.4 | 2.6 | 6.6 | 3.3 | 5.4 | 2.2 |

OH=orthostatic hypotension; POTS=postural tachycardia syndrome; SD=standard deviation; HUT=head-up tilt test; HR=heart rate; SBP=systolic blood pressure; DBP=diastolic blood pressure; MBP=mean blood pressure; PRT=pressure recovery time; Phase IIE=early phase II; Phase IIL=late phase II; KOGS=Korean version of the orthostatic grading scale; OH=orthostatic hypotension; POTS=postural tachycardia syndrome.

^ab^ p < 0.05, OH vs. POTS, one-way ANOVA and Tukey's post-hoc analysis

^abc^ p < 0.05, OH vs. POTS vs. normal HUT, one-way ANOVA and Tukey's post-hoc analysis

^ab, ac^ p < 0.05, OH vs. POTS and OH vs. normal HUT, one-way ANOVA and Tukey's post-hoc analysis

^ab, bc^ p < 0.05, OH vs. POTS and POTS vs. normal HUT, one-way ANOVA and Tukey's post-hoc analysis
